# Kaposi Varicelliform Eruption in a Patient with Pemphigus Vulgaris: A Case Report and Review of the Literature

**DOI:** 10.1155/2020/6695342

**Published:** 2020-12-30

**Authors:** Bibisha Baaniya, Sudha Agrawal

**Affiliations:** Department of Dermatology and Venerology, B.P. Koirala Institute of Health Sciences, Dharan, Nepal

## Abstract

Haemorrhagic crusted lesions over pre-existing pemphigus vulgaris erosions should arouse suspicion of Kaposi varicelliform eruption (KVE). Immediate treatment with antivirals helps to prevent mortality and morbidities. Here, we report a case of a 67-year-old male who developed haemorrhagic crusted lesions on pre-existing pemphigus lesions during his hospital stay and obtained almost 90% resolution of cutaneous lesions of Pemphigus vulgaris as well as Kaposi varicelliform eruption within 2 weeks of acyclovir treatment along with the continuation of systemic steroids. We also highlight the review of the literature of other reported cases with its management.

## 1. Introduction

Pemphigus vulgaris (PV) is an autoimmune intraepidermal blistering disorder of the skin and mucous membrane caused due to damage to intercellular adhesion structures, i.e., desmogleins resulting in acantholysis [1]. Kaposi varicelliform eruption (KVE) is a rare and potentially fatal viral infection typically due to herpes simplex virus (HSV) 1 or 2, rarely by coxsackie A16 virus and vaccinia virus over a pre-existing dermatosis [2]. This phenomenon has been well studied in atopic dermatitis; hence, alternatively, KVE is also called eczema herpeticum. However, it is only rarely known to occur in patients with pemphigus vulgaris [3].

The mechanisms underlying the pathogenesis of KVE remain incompletely understood. It is assumed that the defective skin barrier, acting in conjunction with immune deficiencies (both cell-mediated and humoral immunity), is implicated [4]. Furthermore, treatment of these patients with systemic immunosuppressants also compromises the integrity of the immune system [3].

Antiviral therapy with acyclovir is the treatment of choice, which is usually combined with a systemic antibiotic to control heavy bacterial colonization [5]. Before the advent of acyclovir therapy, the mortality in cases of KVE was as high as 50%; now with specific acyclovir treatment, it is around 10% [6]. Here, a case of pemphigus vulgaris with KVE is being presented along with the review of the literature of other reported cases with its management.

## 2. Patient Information

**Figure 1:**
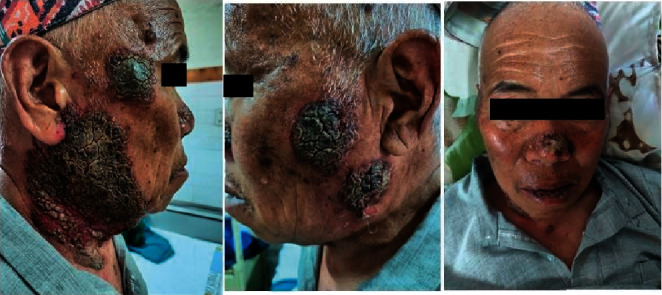
8 months prior to admission: hyperkeratotic plaque and erosions.

A 67-year-old male had presented to Dermatology OPD of our hospital 8 months back with a history of oral erosions for 2 months and cutaneous erosions for 1 month and was diagnosed as pemphigus vulgaris (Figure 1). The patient was advised to take an oral steroid 60 mg daily along with other supportive treatments. In spite of proper counselling of the prognosis and long-term follow-up of pemphigus, he was taking prednisolone on and off and was unable to come for the follow-up because of the COVID-19 pandemic.

### 2.1. Investigations and Treatment

The patient presented again 5 weeks before the admission with multiple erosions that extended to the anterior chest and was advised to continue prednisolone 50 mg once daily, azathioprine (100 mg once daily), doxycycline (100 mg once daily), and nicotinamide (500 mg daily) for 5 weeks.

**Figure 2:**
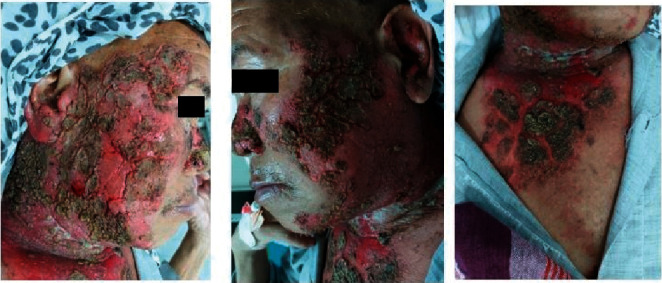
Day 1 of admission: extension of erosions despite high-dose oral steroids.

**Figure 3:**
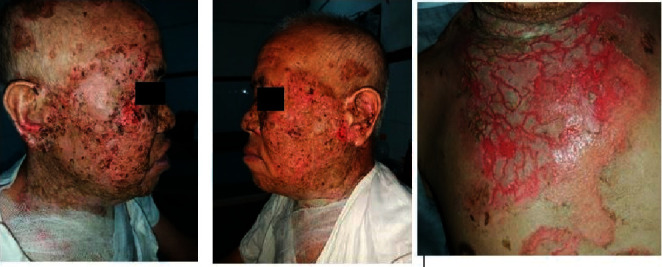
Day 20 of admission: improvement of erosions but appearance of haemorrhagic crusts.

**Figure 4:**
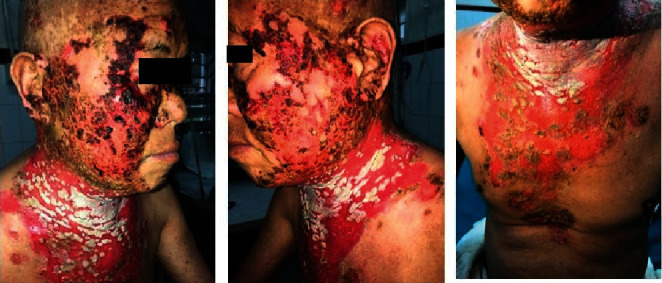
Day 23 of admission: extensive haemorrhagic crusts in the face and few in the periphery of erosions in the chest.

However, he failed to show satisfactory improvement (Figure 2), and his swab for bacterial culture and sensitivity revealed *Pseudomonas aeruginosa* sensitive to piperacillin; hence, he was admitted to the dermatology ward and started on the same antibiotic and intravenous, dexamethasone 8 mg once daily along with skin care. After receiving these medications for 2 weeks, once the pseudomonas infection resolved, dose of dexamethasone was increased to 16 mg over the next 2 weeks, and the patient got significant improvement. However, 1 week after initiating steroid dose escalation, the patient developed few monomorphic haemorrhagic crusts on the cheeks overlying the pre-existing lesions (Figure 3), which progressed over the next week to involve bilateral cheeks, malar region, and bridge of the nose with few lesions in the forehead, bilateral ears, and chest. These lesions were associated with extensive cutaneous pain and myalgia (Figure 4).

**Figure 5:**
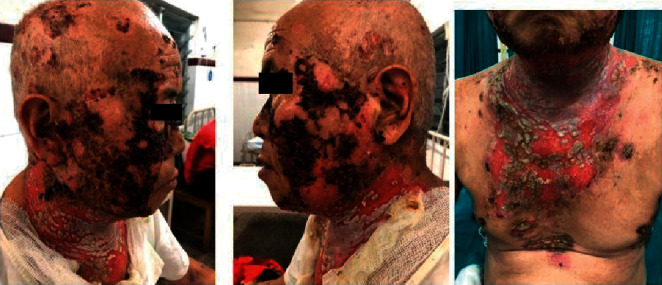
Day 27 of admission: further extension of haemorrhagic crusts.

Although he denied past history of herpes infection, his HSV-1 IgM and IgG also came out to be positive and hence was diagnosed as KVE. Then, he was immediately started on oral acyclovir 400 mg thrice daily, and dexamethasone was tapered rapidly to 9 mg from 16 mg once daily over a week (Figure 5). Further ophthalmological consultation was done, and herpes keratitis was ruled out. Within 1 week of the treatment, the crusts decreased significantly, and treatment was continued. The erosions also healed rapidly thereafter.

His comorbidities were type 2 diabetes mellitus, grade 2 benign prostatic enlargement, right nephrolithiasis, and osteopenia.

### 2.2. Outcome and Follow-Up

**Figure 6:**
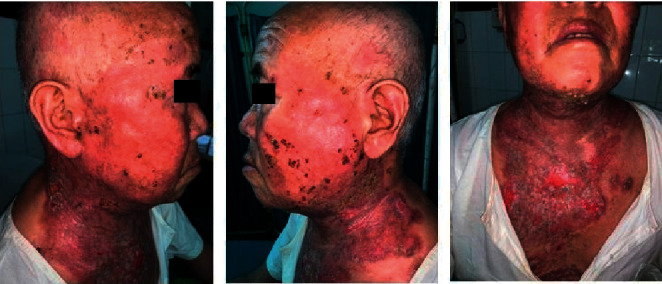
90% resolution of lesions within 2 weeks of commencing oral acyclovir.

**Figure 7:**
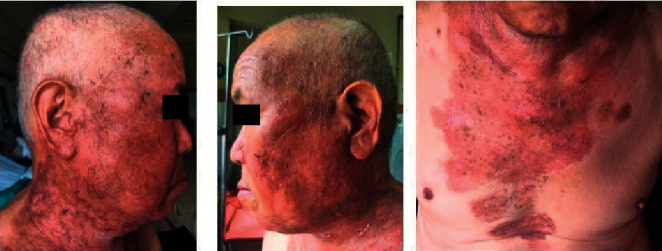
Almost 100% improvement within 4 weeks of commencing antiviral treatment.

Once there was 90% resolution in the haemorrhagic crust, the patient was discharged on 60 mg prednisolone and oral acyclovir 400 mg thrice daily (Figure 6). Oral acyclovir was continued with the same dose for a total of 4 weeks until almost 100% improvement was achieved (Figure 7). After that, monthly tapering of the dose of oral prednisolone was done, and the patient was instructed to follow up via teledermatology consultation during the COVID-19 pandemic as he came from remote hilly area.

## 3. Discussion

Austrian dermatologist Moriz Kaposi first described KVE or eczema herpeticum. Some authors, however, define these two terms differently: eczema herpeticum as disseminated HSV infection over pre-existing eczematous skin disease and KVE as any disseminated cutaneous infection with HSV type 1 or 2 [5].

After an incubation period of about 3–10 days, KVE presents with disseminated eruption of closely grouped, painful, monomorphic, umbilicated vesicles, accompanied by fever, malaise, and regional lymphadenopathy. The vesicles tend to evolve rapidly to pustules or dry out, forming crusts over eroded areas during the course of the disease. The eruption is frequently located on the head, neck, and the upper part of the trunk and usually heals within 2 to 6 weeks [2]. In our case, one week prior to developing haemorrhagic crusts over pre-existing erosions, another patient residing next to our patient had developed herpes labialis and was started on oral acyclovir 400 mg thrice daily. On inquiring, our patient denied any previous herpes infection; hence, we concluded it to be the primary infection he had acquired from the nearby patient. We could not keep our patient in isolation due to logistic constraints secondary to ongoing COVID-19 pandemic.

Recurrent episodes of KVE may occur [7]. Secondary bacterial infection, viremia, and multiorgan involvement are the important causes for mortality; and hence, KVE is a dermatological emergency. Ocular complications such as keratitis, conjunctivitis, blepharitis, uveitis, and loss of vision can also occur [2]. In our patient, herpes keratitis was ruled out after ophthalmological consultation. Here, we list the case series and reports of KVE with pemphigus vulgaris (Table 1).

Kaposi's varicelliform eruption is diagnosed clinically, and investigations such as Tzanck smear and HSV serology are only supportive [2, 3]. Viral culture, direct fluorescence antibody staining, and polymerase chain reaction are the most reliable techniques for herpes simplex virus detection. Histological findings such as intraepidermal blister, acantholysis, ballooning degeneration of the keratinocytes, and multinuclear giant cells with intranuclear inclusion help in diagnosis of HSV infection [7]. In our patient, HSV-1 IgG and IgM both were positive which further supported diagnosis, but other investigations were not done due to logistic constraints.

**Figure 8:**
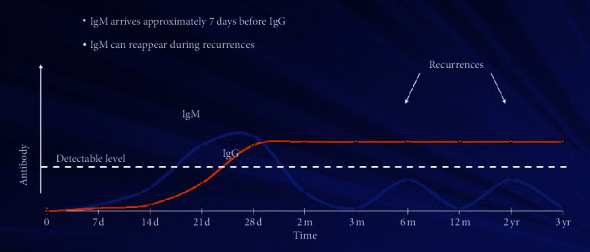
Graph of IgM and IgG response in HSV infection [15].

A positive serology indicates present or past infection; IgM antibody testing cannot discriminate primary versus recurrent episodes of HSV infection, and a positive HSV IgG antibody serology cannot be used for diagnosis of an active infection (Figure 8). If both HSV IgM and IgG are positive, then it implies that infection date is indeterminate [15]. Thus, HSV serology only contributes as a supportive diagnostic tool [2]. In our patient, HSV-1 IgG and IgM both were positive on the 3rd week of exposure with the nearby patient which further supported the fact that he had primary infection.

Treatment of KVE must be initiated with antivirals as soon as possible since it is a potentially life-threatening disease. No formal guidelines for antiviral treatment are established for treatment of KVE in immunobullous diseases, and different authors have followed different guidelines (Table 1). In a study by Lehman and el-Azhary, they used valacyclovir 1 g PO BD for 10 days or acyclovir 400 mg PO TDS for 10 days or ganciclovir during the first episode. In recurrent episodes, they initiated valacyclovir 1 g PO OD or acyclovir 400 mg PO BD as chronic suppressive therapy [3]. Zouhair et al. continued oral acyclovir until complete lesion resolution in herpetic superinfection of the pemphigus patient, and they observed relief in 5 to 20 days [16]. Similarly, our case achieved almost 100% resolution of lesions with 4-week acyclovir therapy. On the contrary, Rao et al. treated their patients with oral acyclovir 800 mg 5 times a day for 10 to 14 days [9]. The prodrug valacyclovir is converted into acyclovir during 1st-pass metabolism in the liver but has a better oral bioavailability than acyclovir [17].

Lehman and el-Azhary also considered reducing the dose of systemic immunosuppressants and claimed that patients with severe skin disease or with additional complications from the viral infection may require intravenous antiviral therapy [3]. In our patient, we continued oral acyclovir 400 mg thrice daily until complete resolution, i.e., 4 weeks, and dose of the systemic steroid was also decreased with significant improvement in patient's symptoms.

Although we have tried our best to manage our patient, lack of PCR and immunological tests such as direct immunofluorescence, indirect immunofluorescence, and ELISA due to logistic constraints are the major limitations.

### 3.1. Patient's Perspective

When I developed oral and skin erosions, I immediately consulted the doctors and was prescribed some oral and topical medications. However, they failed to improve the lesions which really made me frustrated. Nevertheless, I always had trust in my doctor, and as per their suggestion, I got admitted to ward. After that, my lesions healed significantly, and now, I am happy that almost all of my erosions are gone.

## 4. Conclusion

The diagnosis of Kaposi varicelliform eruption is usually made clinically. Appearance of umbilicated vesicular rash is the usual presentation, but haemorrhagic crusts over pre-existing lesions are an uncommon presentation as in our case. Investigations such as PCR, viral culture, histopathology, HSV serology, and Tzanck smear have only supportive value. It is also important to diagnose it in time and start antiviral treatment immediately as it is associated with significant morbidity and mortality. Acyclovir 400 mg PO TDS or 10 mg/kg IV or valacyclovir 500 mg PO BD until resolution of KVE is usually recommended. Similarly, the dose of corticosteroids can be decreased or stopped depending on the clinical status of pemphigus vulgaris and other immunosuppressants such as mycophenolate mofetil can be deferred until resolution of KVE.

## Figures and Tables

**Table 1:** Case series and reports of KVE with pemphigus vulgaris.

SN	Author/year	Age (years)/sex	Morphology of pemphigus vulgaris	Morphology of KVE	Investigations	Treatment of KVE	Treatment of pemphigus vulgaris	Outcome of KVE
1	Lehman and el-Azhary [3]	44.1/male	Focal lesions with oral involvement	Not mentioned	Polymerase chain reaction (PCR), skin swabs, viral culture, direct immunofluorescence	Ganciclovir (PO) (dose and duration not mentioned)	Mycophenolate mofetil, prednisone (modification not mentioned)	Resolved (time to resolution not mentioned)
		51.6/female	Widespread lesions with oral involvement	Not mentioned	PCR, skin swabs, viral culture, direct immunofluorescence	Acyclovir (IV) (dose and duration not mentioned)	Intramuscular corticosteroids (modification not mentioned)	Resolved (time to resolution not mentioned)
		70.2/female	Focal lesions with oral, conjunctival, perianal involvement	Not mentioned	PCR, skin swabs, viral culture, direct immunofluorescence	Valacyclovir hydrochloride 1 gm PO BD × 10 days	Azathioprine, prednisone (modification not mentioned)	Resolved (time to resolution not mentioned)
		39.3/female	Focal lesions with oral involvement	Not mentioned	PCR, skin swabs, viral culture, direct immunofluorescence	Acyclovir (IV) (dose and duration not mentioned)	Mycophenolate mofetil, azathioprine, prednisone (modification not mentioned)	Resolved (time to resolution not mentioned)
		45.9/female	Oral erosions only	Not mentioned	PCR, skin swabs, viral culture, direct immunofluorescence	Valacyclovir hydrochloride 1 gm PO BD × 10 days	None	Resolved (time to resolution not mentioned)
		85.7/female	Focal lesions with oral involvement	Not mentioned		Acyclovir (IV) (dose and duration not mentioned)	Rituximab (modification not mentioned)	Resolved (time to resolution not mentioned)

2	Nath et al. [8]	40/female	Not mentioned	Lesions in the face, neck, trunk, upper limbs, and thighs	Tzanck smear positive	Acyclovir 400 mg PO TDS × 5 days	DCP (phase 1, 1st cycle) (modification not mentioned)	KVE not healed and PV worsened, the patient left the hospital in critical condition
		27/female	Not mentioned	Lesions in breasts	Tzanck smear positive	Acyclovir 400 mg PO TDS × 10 days	Dexamethasone cyclophosphamide pulse (DCP) (phase 1, 3rd cycle), prednisolone, cyclophosphamide (modification not mentioned)	KVE resolved, PV unaltered (time to resolution not mentioned)
		26/female	Not mentioned	Lesions in the trunk, upper limbs, and thighs	Tzanck smear positive	Acyclovir 500 mg IV TDS × 18 days	Dexamethasone azathioprine pulse (DAP) (phase 1, 1st cycle), prednisolone, azathioprine (modification not mentioned)	KVE partially healed, the patient left the hospital
		40/female	Not mentioned	Lesions in the trunk	Tzanck smear positive	Acyclovir 400 mg PO TDS × 13 days	DCP (phase 1, 3rd cycle), prednisolone, cyclophosphamide (modification not mentioned)	KVE resolved, PV unaltered (time to resolution not mentioned)

3	Rao et al. [9]	30/male	Not mentioned	Umbilicated grouped vesicular eruption around the eyes, mouth, and axilla	Tzanck smear positive, IgM positive	Acyclovir 800 mg PO 5 times a day × 14 days	DCP (modification not mentioned)	Resolved (time to resolution not mentioned)
		45/female	Not mentioned	Umbilicated grouped vesicular eruption around the eyes, mouth, and axilla	Tzanck smear positive, IgM positive	Acyclovir 800 mg PO 5 times a day × 14 days	DCP (modification not mentioned)	Succumbed to multiorgan failure

4	Vora et al. [7]	26/male	Old crusted lesions of PV over the scalp with patchy hair loss	Umbilicated vesicular lesions over the face, chest, back, and limbs	Tzanck smear positive, histopathology suggestive of KVE	Acyclovir 10 mg/kg IV every 8 hours × 10 days	Prednisolone 10 mg, azathioprine 50 mg BD (no modification)	Healed with varicelliform scars (time to resolution not mentioned)

5	Marfatia et al. [10]	30/male	Erosions in the oral cavity, genitals, and trunk	Not mentioned	HIV positive, chest X-ray suggestive of pulmonary TB	Acyclovir (PO) (dose and duration not mentioned)	Dexamethasone IM TDS, treatment of tuberculosis and HIV (modification not mentioned)	KVE resolved (time to resolution not mentioned)

6	Corral et al. [1]	33/male	Not mentioned	Painful bullous lesions on the scalp, oral cavity, and trunk	Not mentioned	Acyclovir (IV) (dose and duration not mentioned)	Intravenous immunoglobulin, followed by a second pulse therapy with methylprednisolone, mycophenolate mofetil was held	KVE resolved (time to resolution not mentioned)

7	Feldmeyer et al. [11]	71/male	Not mentioned	Refractory vegetating skin lesions, especially of the centrofacial area	Nasal smear was positive for HSV2 by direct immunostaining, culture negative	Valacyclovir 500 mg PO BD × 10 days	Prednisone, and cyclosporine, intravenous immunoglobulin (modification not mentioned)	KVE resolved in 10 days and PV also remitted

8	Chiu et al. [12]	66/female	Not mentioned	Generalized painful lesions over the lower trunk, buttocks, bilateral popliteal, and inguinal areas	Histopathology and immunohistochemistry for herpes virus were positive; HSV IgG increased	Famciclovir 750 mg PO OD × 10 days	Discontinued the systemic steroid	KVE showed remarkable improvement in 10 days

9	Mackley et al. [13]	72/female	Not mentioned	Innumerable deep, punched-out erosions scattered over the existing geographic erosions	Tzanck smear positive, direct fluorescent antibody test positive for HSV-1	Acyclovir 15 mg/kg IV OD (duration not mentioned); discharged on chronic prophylactic acyclovir	Prednisone, mycophenolate mofetil, and cyclosporine (modification not mentioned)	Significant improvement (time to resolution not mentioned)

10	Ortiz [14]	66/male	Not mentioned	Lesions in the chin, cheeks, and neck	Tzanck negative, biopsy suggestive of KVE	Not mentioned	Prednisone, gold salts (modification not mentioned)	Not mentioned

11	Our case (2020)	67/male	Erosions in the face, neck, chest, and oral cavity	Haemorrhagic crusts in the face, neck, and chest over pre-existing erosions	HSV-1 IGM and IgG positive	Acyclovir 400 mg PO TDS × 4 weeks	Dexamethasone decreased from 16 mg to 9 mg, azathioprine continued	Almost 100% resolution in both KVE and PV within 4 weeks
